# Cost-effectiveness of primary offer of IVF vs. primary offer of IUI followed by IVF (for IUI failures) in couples with unexplained or mild male factor subfertility

**DOI:** 10.1186/1472-6963-6-80

**Published:** 2006-06-23

**Authors:** Nora Pashayan, Georgios Lyratzopoulos, Raj Mathur

**Affiliations:** 1 Department of Public Health and Primary Care, Institute of Public Health, University Forvie Site, Robinson Way, Cambridge CB2 2SR, UK; 2 Norfolk Suffolk and Cambridgeshire Strategic Health Authority, Victoria House, Capital Park, Fulbourn, Cambridge, CB1 5XB, UK; 3 Cambridge University Teaching Hospitals Foundation Trust, Hills Road, Cambridge, CB2 2QQ, UK

## Abstract

**Background:**

In unexplained and mild male factor subfertility, both intrauterine insemination (IUI) and in-vitro fertilisation (IVF) are indicated as first line treatments. Because the success rate of IUI is low, many couples failing IUI subsequently require IVF treatment. In practice, it is therefore important to examine the comparative outcomes (live birth-producing pregnancy), costs, and cost-effectiveness of primary offer of IVF, compared with primary offer of IUI followed by IVF for couples failing IUI.

**Methods:**

Mathematical modelling was used to estimate comparative clinical and cost effectiveness of either primary offer of one full IVF cycle (including frozen cycles when applicable) or "IUI + IVF" (defined as primary IUI followed by IVF for IUI failures) to a hypothetical cohort of subfertile couples who are eligible for both treatment strategies. Data used in calculations were derived from the published peer-reviewed literature as well as activity data of local infertility units.

**Results:**

Cost-effectiveness ratios for IVF, "unstimulated-IUI (U-IUI) + IVF", and "stimulated IUI (S-IUI) + IVF" were £12,600, £13,100 and £15,100 per live birth-producing pregnancy respectively. For a hypothetical cohort of 100 couples with unexplained or mild male factor subfertility, compared with primary offer of IVF, 6 cycles of "U-IUI + IVF" or of "S-IUI + IVF" would cost an additional £174,200 and £438,000, representing an opportunity cost of 54 and 136 additional IVF cycles and 14 to 35 live birth-producing pregnancies respectively.

**Conclusion:**

For couples with unexplained and mild male factor subfertility, primary offer of a full IVF cycle is less costly and more cost-effective than providing IUI (of any modality) followed by IVF.

## Background

In any health care system, cost-effective commissioning of health care in order to maximise population health outcomes with the minimum possible resource use, is an important consideration. The UK National Institute for Clinical Excellence (NICE) [1] recommends offer of up to six cycles of Unstimulated Intrauterine Insemination (U-IUI) for couples with unexplained or mild male factor subfertility. Supplementary cost calculation guidance suggested overall savings to the NHS from a "switch" of activity from Stimulated IUI (S-IUI) to U-IUI, which was also recommended [2]. These calculations however were based on 'head-to-head' comparisons between S-IUI and U-IUI and completely ignored the cost implications of the fact that many couples who fail IUI will subsequently require in-vitro fertilisation (IVF) treatment.

Even if some health care interventions may have a relatively low cost per treatment session, they could be less cost-effective compared to alternative treatments with a higher cost per treatment session, but also better clinical effectiveness. It is therefore legitimate to examine critically whether funding for IUI allows better management of current demand, or whether it increases activity and overall associated healthcare expenditure for those couples who are eligible for both treatment strategies [3]. We therefore constructed a mathematical model to evaluate the cost-effectiveness of provision of IUI followed by IVF compared to primary offer of IVF for patients who according to NICE are eligible for both treatment strategies.

## Methods

### Model construction

**Figure 1:**
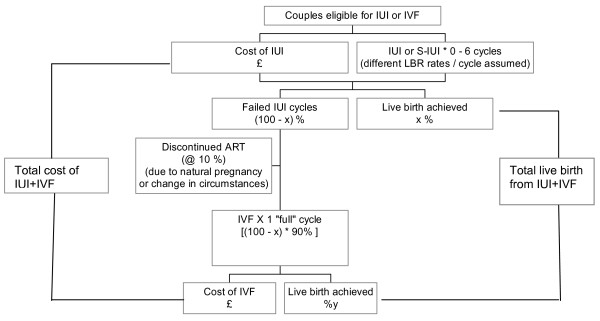
**The structure of the generic model.** U-IUI: Unstimulated IUI S-IUI: Gonadotrophin stimulated IUI C-IUI: Clomifene stimulated IUI ART: Assisted reproductive technique LBR: Live birth rate

The structure of the generic model is presented in Figure 1. The model emulates the clinical experience of a hypothetical cohort of 100 couples with unexplained or mild male factor subfertility by different treatment strategy, including, when applicable, the transition from one to another treatment stage or strategy. The outcome examined was live birth-producing pregnancy. Three different scenarios were used sequentially, relating to comparisons of primary offer of IVF with three possible modalities of IUI: U-IUI, S-IUI (gonadotrophin-stimulated) and C-IUI (clomifene-stimulated). Each of the three scenarios allowed for estimation of activity and outcomes for offer of up to six IUI cycles, as per NICE guidance [1].

The EVEREST checklist for health economic studies was used as a reference to our study design (Additional File 1).

### Assumptions used in the model

Assumptions used in the calculations are presented in Table 1. Evidence to populate the models was derived from peer-reviewed literature and, in the absence of such literature data, based on activity data of local subfertility unit. For estimates from the literature, we have used the excellent systematic review conducted by NICE to underpin their 2004 guidance on subfertility treatments. In addition, we have searched MEDLINE for any more recent studies on live birth rate (LBR) of S-IUI, U-IUI, and C-IUI. Relevant studies are referenced in relevant parts of the manuscript.

A cycle of IVF included a fresh cycle, and when applicable, (i.e. in couples who had additional viable embryos preserved at the time of the fresh embryo transfer, and subsequently did not achieve pregnancy through the fresh cycle) frozen embryo transfer (FET) cycles. All estimates assume a "steady state" between incident need and supply of services, and a "steady state" in progression (e.g. waiting or natural lag times) for transition to a subsequent treatment stage or strategy.

Average LBR values for fresh as well as FET IVF cycles were derived from the UK's Human Fertilisation and Embryology Authority (HFEA) population-based register 1995 to 1999 as used by NICE [1]. As supported by evidence, it was assumed that the average LBR derived from HFEA data is applicable to couples accessing IVF after previous failed IUI attempts.

Unlike IVF, there is no population-based data on the effectiveness (LBR) of S-IUI. Therefore, two sets of values for LBR were considered either based on, or informed by, data derived from the experience of a local unit, which during the period of 1993 to 2003 provided 676 cycles of S-IUI to 334 different women aged < 40 years of age, and with a range of uptake of 1 to 5 cycles (Additional File 2) [5]. Firstly, an average LBR value for S-IUI of 7% per cycle, independently of treatment cycle order was used. Secondly, LBR values of 10%, 6%, 4%, 4%, 2%, and 2% for the 1^st ^to the 6^th ^cycle respectively were used. The first scenario (mean effectiveness independent of cycle order) is reasonable, as small numbers did not allow for confident calculation of LBR for subsequent to the first cycle. The second scenario reflects both the actual experience of the local unit [5], and peer-reviewed evidence [6-10], indicating decreasing effectiveness of IUI with order of cycle.

Unlike S-IUI, U-IUI has, at least up until the publication of the NICE guidelines [1] in 2004, been practiced very infrequently in the UK, therefore, there were no national or local data of relevance to U-IUI outcomes. Given this, the LBR of U-IUI was estimated to be half of that of S-IUI [11]. Similarly, the LBR for C-IUI was assumed to be 3%, i.e. 60% lower than of S-IUI, as suggested by a Cochrane review [12].

It was assumed that on average 10% of couples who fail IUI treatment (irrespective of the number of IUI cycles received) would drop out of subfertility treatment. This was based on informed judgement, to reflect potential changes in childbearing intentions or partnership status.

### Cost calculations

Costs relate to resources directly associated with fertility treatment, and exclude costs from potential complications and/or multiple pregnancy. The average costs per cycle of IVF, FET, U-IUI, and S-IUI used were the ones used in NICE's economic model [1], and were consistent with NHS costs for these treatments locally. In the absence of a relevant information in NICE's economic model, it was assumed that the cost of C-IUI is approximately 'half way' between cost of U-IUI and S-IUI (£752), based on local expert opinion and knowledge of drug costs.

### Cost-effectiveness

The perspective used in the cost-effectiveness analysis was that of the health service. The total cost to the health service was taken as the sum of the costs of the IUI and the IVF activity generated by each scenario.

Incremental cost-effectiveness ratio (ICER) was calculated as the ratio of the difference in the combined cost of "IUI + IVF" strategies minus the cost of IVF, divided by the difference in number of live birth-producing pregnancies for each treatment strategy. In this instance ICER values therefore indicate the additional cost associated with a given treatment strategy in order to produce a single additional live birth-producing pregnancy.

### Sensitivity analysis

Sensitivity analysis was undertaken to examine the robustness of the modelling and to study the impact of plausible variation in the variables for which uncertainty was judged to be higher (e.g. those least well supported by population-based data or peer-reviewed publications). Therefore, the estimates of U-IUI, S-IUI and C-IUI were varied sequentially, using different value estimates from the literature, or in the absence of alternative literature estimates, based on local experience, expert opinion, or reasonable assumptions about the plausible range of uncertainty around "best estimate" values. More specifically:

#### • for S-IUI

An alternative LBR estimate of 8.7% per cycle (independently of cycle order) was used based on Goverde et al [13].

#### • for U-IUI

An alternative LBR estimate of 4.4% per cycle (independently of cycle order) was used, assuming the LBR of U-IUI to be half of that of S-IUI [11]. In addition, LBR values of 5%, 3%, 2%, 2%, 2%, 2% for the 1^st ^to the 6^th ^cycle respectively were also used for U-IUI, again assuming the effectiveness of U-IUI to be half of that of S-IUI [11]. Lastly, a third estimate of an average LBR of 2% per cycle was used based on local clinical experience and expert opinion.

#### • for C-IUI

An alternative LBR estimate of 6.6% per cycle (independently of cycle order) was used based on reasonable assumption of having LBR of C-IUI in between LBR values of S-IUI and U-IUI. Additionally, an average LBR value of 2.5% per cycle was used, based on local experience and expert opinion.

## Results

For a hypothetical cohort of 100 couples with unexplained or mild male factor subfertility, the cost of primary offer of one full cycle of IVF would be £321,700, with a cost-effectiveness ratio of £12,600/live birth-producing pregnancy (Table 2).

### Cost-effectiveness of 'head-to-head' comparison of primary IUI vs. primary IVF

Primary offer of one to six cycles of U-IUI or C-IUI, or up to three cycles of S-IUI is less costly than one cycle of fresh IVF. However, when comparing the cost-effectiveness of U-IUI, S-IUI and C-IUI to one full cycle of IVF, IVF is more cost-effective (Table 2).

### Cost-effectiveness of "IUI + IVF" vs. primary IVF

#### Assuming a mean U-IUI LBR of 3.5% per cycle

For six cycles of U-IUI the cost of IUI followed by IVF would be £495,900 with a cost-effectiveness ratio of £13,100/live birth-producing pregnancy (Table 2). Compared to primary offer of IVF, the ICER for offer of one to six cycles of U-IUI followed by IVF (for IUI failures) would be £18,000 to £14,200 per live birth-producing pregnancy.

#### Assuming a mean S-IUI LBR of 7% per cycle

For one to six cycles of S-IUI per couple, the total cost of "IUI+IVF" would range from £369,000 to £759,800, equivalent to £13,000 to £15,100 per live birth-producing pregnancy. The ICER would increase with increase in cycle order (Table 2).

For one to six cycles of IUI, the "IUI + IVF" treatment strategy would be more costly and less cost-effective than one full cycle of IVF.

**Figure 2:**
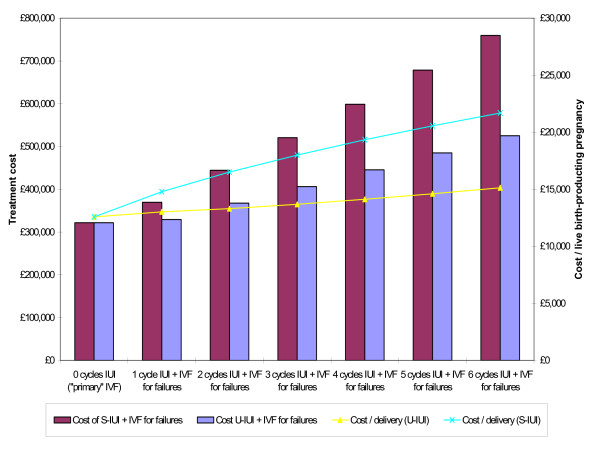
Cost and cost-effectiveness (per live birth-producing pregnancy) of different uptake of IUI and S-IUI among a hypothetical cohort of 100 couples eligible for both IUI and IVF. Assumes constant LBR of 7% and 3.5% for S-IUI and IUI.

Figure 2 compares total cost and cost-effectiveness of U-IUI to S-IUI. U-IUI is more cost-effective than S-IUI at any cycle order.

#### Assuming a mean C-IUI LBR of 3% per cycle

Among the three IUI modalities, C-IUI (with the lowest LBR) had the worst cost-effectiveness ratio and the worst ICER.

When assuming that LBR of IUI (any modality) was decreasing with cycle order, all above scenarios showed consistently similar results, with higher cost and worst cost-effectiveness and ICER values for "IUI+IVF" compared to IVF only.

### Opportunity costs

Table 3 shows the opportunity costs, in terms of number of additional IVF cycles that could be purchased by the cost difference of IUI (followed by IVF) compared to primary offer of IVF alone. Providing six cycles of U-IUI and one full cycle of IVF to 90% of couples who failed IUI, would increase subfertility related health care expenditure by £174,200. If this amount was allocated for purchasing of IVF, then an additional 54 couples would have access to IVF and an additional 14 couples would achieve a live birth-producing pregnancy. If six cycles of S-IUI were offered instead of U-IUI, the otherwise avoidable cost to the health care system would be an additional £438,000, sufficient to otherwise fund 136 IVF cycles, and to enable additional 35 couples to achieve live birth. If a smaller number of IUI cycles is offered the opportunity costs would be minimised, but the net difference would always be in favour of the primary IVF strategy.

### Sensitivity analysis

After considering different plausible LBR values for S-IUI, U-IUI and C-IUI, the primary offer of IVF remained more cost-effective than providing "IUI+IVF" (Table 4) and associated with less opportunity cost (Table 5).

## Discussion

The findings suggest that for couples with unexplained or mild male factor subfertility, primary offer of a full IVF cycle is less costly and more cost-effective than primary offer of any IUI modality followed by IVF (for IUI failures). Importantly, the cost of primary IUI (any modality) increases and its cost-effectiveness decreases with increasing number of IUI cycles offered. Between S-IUI and U-IUI, S-IUI followed by IVF was associated with higher cost and lower cost-effectiveness compared with U-IUI followed by IVF. The sensitivity analysis findings indicate that the main finding of inferior cost-effectiveness of the primary IUI, compared with primary IVF, is robust. A previous study on cost impact implications of the 2004 NICE guidance has failed to reach similar conclusions, as it did not accurately simulate real life patient experience and clinical management [1].

In principle, a prospective randomised controlled trial would have been a superior study design to answer this study's research question. However, such a study would have had to entertain certain practical obstacles, including ethical clearance, and may not be forthcoming for many years to come. At the same time resource allocation and service development policy decisions have to be made currently and in the near future. In the absence of RCT-based evidence, mathematical modelling synthesising evidence from various high quality sources is the only rational way to support health care policy decision-making [14].

Published data comparing the cost-effectiveness of IVF versus IUI are scarce. Some studies indicate that IUI may be more cost-effective than IVF in cases of unexplained and moderate male subfertility [13,15-17]. However, these studies have not accounted for the potential subsequent FET component of IVF strategies, which increases their cost-effectiveness [17]. More importantly, these studies do not take into account the potential for eventual "transfer" of most couples who fail IUI to IVF, a crucial consideration by commissioners of healthcare services when both IUI and IVF are funded from the same budget (as is the case in the UK NHS).

The majority of the literature on IUI effectiveness comes from uncontrolled retrospective case series and cohort designs, could overestimate treatment effectiveness because of selection bias and consideration of clinical pregnancy as opposed to live birth as the study outcome. Also, the number of abandoned cycles is generally excluded from the denominator, leading to inflated estimates of effect [11]. A further problem is that the reported effectiveness of IUI is very variable, ranging from small integer numbers to up to over 30%. This heterogeneity may be due to factors including differences in study populations, ovarian stimulation regimes, the number of inseminations per cycle, the timing of insemination, and methods of sperm preparation [18]. Also, the reported trials involved an exceedingly small fraction of the very great number of patients world-wide undergoing treatment, so generalisations have to be cautious [19]. Given the above limitations of published evidence, we used local IUI activity data as the main source of our data in relation to the effectiveness of IUI, but complemented those with different sets of estimates in sensitivity analysis. The results obtained using local current data could better reflect the likely current UK practice in a "real life" situation, and for an unselected population.

We used a mean LBR value of 7% for S-IUI based on local experience on 676 cycles of S-IUI over 10 years. In most studies the pregnancy rate per cycle of S-IUI is approximately 10% [20-22]. A prospective randomised study from The Netherlands showed that the average LBR of S-IUI of 8.7% per cycle [13]. NICE in its economic model has used results of an RCT from the US [23] reporting cumulative pregnancy rates of 33% for S-IUI for an average of 5.6 cycles. In our model, if the cumulative LBR for five and six cycles of S-IUI is considered, it equates to 30% and 35% respectively, and as such our assumptions are comparable to the order of the estimates used by NICE.

The effectiveness (LBR) of IUI was judged to be 3.5% per treatment cycle -this represents an informed judgement, based on the literature. Many experts would support that this is actually a high estimate, but we preferred to use a "generous" estimate. If the true estimate is lower, any bias introduced from this assumption would have favoured the cost-effectiveness of U-IUI, and would have made IVF less cost effective, i.e. if a lower LBR value for U-IUI was used, the results would have been even more favourable for primary offer of IVF as the preferred treatment strategy.

We used average success rates rather than age- and indication-specific LBRs. Maternal age affects outcomes of IUI, but so does for IVF [1]. Similarly, different subfertility indications are associated with different success rates for IUI, but again the same is true for IVF. Therefore, the lack of stratification (by age or indication) is very unlikely to influences the results for either modality. In any case, the hypothetical cohort of 100 couples can be assumed to have an identical case mix in terms of age and indication stratification, whichever treatment strategy is offered.

The modelling of costs has concentrated on the nominal cost of IVF and IUI. Other essential aspects of the service (e.g. diagnostic activity, incident and follow-up appointments, counselling) have not been calculated, because they were assumed to be already occurring (i.e. requiring no 'new' money) and to be similar to both treatment strategies. The cost associated with complications, such as multiple gestation and birth, were also not included in calculations. Evidence suggests that there is no statistically significant difference in the incidence of ovarian hyperstimulation syndrome (OHSS) and multiple pregnancy rate between S-IUI and IVF [13]. As such, it is unlikely that inclusion of the costs of complications would have had an impact on the comparative cost-effectiveness of S-IUI and IVF.

Lastly, anecdotally, undergoing IUI followed by IVF may be more psychologically distressing to the couple compared to IVF alone, as it may be more frustrating to have, on average, comparatively more failed treatment cycles -although direct evidence about this assumption is lacking. However, some patients may have a positive preference for IUI or an aversion for IVF (on philosophical, or religious grounds). We acknowledge that traditional methods of cost-effectiveness analysis like the ones used in this study, are not useful tools when dealing with philosophically-grounded patient preferences – which however are exceptional, rather than the norm.

## Conclusion

Our study indicates that for couples with unexplained or mild male factor subfertility, a primary treatment offer of any modality of IUI instead of primary offer of IVF is cost-ineffective, and therefore associated with considerable opportunity costs. The additional and avoidable costs incurred put pressure on the health care system to cope with extra demand and activity for a treatment of low effectiveness, making the wider availability of the most effective treatment (IVF) more difficult, and thus disadvantaging couples who could have otherwise benefited from it.

## Competing interests

The author(s) declare that they have no competing interests.

## Authors' contributions

GL and NP constructed the mathematical model, reviewed the literature on estimates of treatment effectiveness, and inputted data into the model. RM collected and provided original data on IUI effectiveness used in the model and helped with literature search and interpretation. All authors contributed to the writing of the manuscript. GL is the guarantor.

## Pre-publication history

The pre-publication history for this paper can be accessed here:



## Supplementary Material

Additional File 1 EVEREST Statement: Checklist for health economics paper. EVEREST checklist for health economics studies with respect to this study design, data collection, and analysis and interpretation of the results Click here for file

Additional File 2 Local data on outcome of 676 cycles of S-IUI in 334 couples with unexplained infertility women aged <40 years. Results of local data on S-IUI uptake and outcome, live birth-producing pregnancy, by treatment cycle order. Click here for file

## Figures and Tables

**Table 1:** Assumptions underlying the modelling and their corresponding sources

**Assumption**	**Source**
There is an 1:1 ratio between "fresh" and "frozen" cycles, assuming 25% of couples have 0 FET, 50% have 1 FET and 25% have 2 FET	Hypothetical assumption, based on informed judgement
IVF and IVF/ICSI ratio is 1:1	Hypothetical assumption, based on informed judgement
100% compliance and 0% drop out with IUI treatment	Hypothetical assumption, based on informed judgement

**Effectiveness**	

LBR following a "full" IVF (IVF/ICSI) cycle is 19.26% (all ages)	HFEA 1995–1999^1^
LBR following "frozen" IVF (IVF/ICSI) cycle is 10.40% (all ages)	HFEA 1995–1999^1^
LBR following unstimulated IUI is 3.5% per cycle	Informed judgement, based on local data on effectiveness of stimulated IUI and by halving this, peer-reviewed literature^11^
LBR following gonadotrophin-stimulated IUI is 7% per cycle	Based on local activity data
LBR following clomifene- stimulated IUI is 3%	Informed judgement, local activity data, and peer reviewed literature ^12^

**Cost**	

-Cost of "fresh" IVF (IVF/ICSI) is £3,214 assuming 50% of "fresh" cycles will be ICSI cycles -Cost of "frozen" IVF (IVF/ICSI) is £450 -Cost of unstimulated IUI is £449 -Cost of stimulated IUI is £1005 -Cost of Clomiphene stimulated IUI is £752	NICE estimates^1^
90% of couples who fail IUI proceed to IVF	Hypothetical, based on informed judgement

LBR: Live birth rate

FET: Frozen embryo transfer

**Table 2:** Cost, outcome, cost-effectiveness ratio and incremental cost-effectiveness ratio (ICER) of providing unstimulated IUI (U-IUI), gonadotrophin stimulated IUI (S-IUI) and clomifene stimulated IUI (C-IUI) to a hypothetical cohort of couples with unexplained or male factor subfertility.

**U-IUI (LBR = 3.5%)**					**Full cycle of IVF**			**Overall**			
**Order of cycle**	**No. of cycles**	**Live- birth Deli-very from IUI**	**Cost £**	**Cost/outcome (£/delivery)**	**No. of couples having one full cycle of IVF**	**Live birth delivery from one full cycle of IVF**	**Cost £**	**Total live birth delivery (IUI+ IVF)**	**Total cost £**	**Total cost/total outcome (£/Delivery)**	**ICER**

**U-IUI (LBR-3.5%)**											

**0**	0	0	0		100	26	321,700	26	321,700	12,600	Ref
**1**	100	4	44,900	12,800	87	22	279,400	26	324,300	12,700	18,600
**2**	196	7	88,200	12,800	84	21	269,600	28	357,900	12,600	13,200
**3**	293	10	131,600	13,000	81	21	260,200	31	391,800	12,700	13,400
**4**	390	13	175,000	13,200	78	20	251,100	33	426,100	12,800	13,600
**5**	487	16	218,500	13,400	75	20	242,300	36	460,800	13,000	13,900
**6**	584	19	262,000	13,600	73	19	233,800	38	495,900	13,100	14,200
											

**S-IUI (LBR = 7%)**											

**0**	0	0	0		100	26	321,700	26	321,700	12,600	Ref
**1**	100	7	100,500	14,400	84	21	269,300	28	369,800	13,000	24,000
**2**	193	14	194,000	14,400	78	20	250,400	33	444,400	13,300	17,500
**3**	286	20	287,900	14,700	72	19	232,900	38	520,800	13,700	16,600
**4**	380	25	382,300	15,200	67	17	598,900	42	598,900	14,100	17,300
**5**	475	30	477,200	15,700	63	16	678,600	46	678,600	14,600	17,800
**6**	570	35	572,400	16,200	58	15	759,800	50	759,800	15,100	18,300
											

**C-IUI (LBR = 3%)**											

**0**	0	0	0		100	26	321,700	26	321,700	12,600	Ref
**1**	100	3	75,200	25,100	87	22	280,900	25	356,000	14,000	*
**2**	197	6	148,100	24,700	85	21	272,400	28	420,600	15,300	49,400
**3**	294	9	221,200	24,600	82	21	264,000	30	485,200	16,300	40,900
**4**	391	12	294,200	24,500	79	20	255,600	32	549,700	17,200	38,000
**5**	488	15	367,200	24,500	77	20	247,100	34	614,300	17,900	36,600
**6**	585	18	440,200	24,500	74	19	238,700	37	678,900	18,600	32,500

* It was not possible to estimate a meaningful ICER (ICER = (£356073-£321733)/(25.3–25.6) = -£114,467/Live birth-producing pregnancy as compared to full cycle of IVF).

Cost/unit; IVF = £3,214, U-IUI = £449, S-IUI = £1,005, C-IUI = £752Live birth delivery refers to live birth- producing pregnancyFigures are rounded to the nearest hundred

**Table 3:** Cost difference, number of additional full cycles of IVF that could be purchased and corresponding additional number of live birth-producing pregnancies by the estimated cost difference of providing "IUI+IVF" compared to primary offer of IVF

	**"IUI + IVF" for those failing IUI**					
**IUI modality**	**One cycle of IUI**	**Two cycles of IUI**	**Three cycles of IUI**	**Four cycles of IUI**	**Five cycles of IUI**	**Six cycles of IUI**

**U-IUI (LBR = 3.5%)**						

Cost difference "IUI+IVF" vs. primary IVF	£2,600	£36,100	£70,100	£104,400	£139,100	£174,200
N^o ^of IVF cycles that would be purchased by the cost difference	1	11	22	32	43	54
N^o ^of live birth-producing pregnancies expected from IVF cycles that would have been purchased by the cost difference	0	4	6	8	11	14

**S-IUI (LBR = 7%)**						

Cost difference "IUI+IVF" vs. primary IVF	£48,100	£122,600	£199,100	£277,200	£356,900	£438,000
N^o ^of IVF cycles that would be purchased by the cost difference	15	38	62	86	111	136
N^o ^of live birth-producing pregnancies expected from IVF cycles that would have been purchased by the cost difference	4	10	16	22	28	35

**C-IUI (LBR = 3%)**						

Cost difference "IUI+IVF" vs. primary IVF	£34,300	£98,900	£163,400	£228,000	£292,600	£357,200
N^o ^of IVF cycles that would be purchased by the cost difference	11	31	51	71	91	111
N^o ^of live birth-producing pregnancies expected from IVF cycles that would have been purchased by the cost difference	3	8	13	18	23	28

Figures are rounded to the nearest hundred.

**Table 4:** Sensitivity analysis estimates of incremental cost-effectiveness ratios (ICER) of providing "IUI+IVF" compared to primary offer of IVF, varied sequentially.

**Varied variables**	**Source of assumption**	**ICER (£/live birth-producing pregnancy)**					
		**1 cycle of IUI**	**2 cycles of IUI**	**3 cycles of IUI**	**4 cycles of IUI**	**5 cycles of IUI**	**6 cycles of IUI**

**S-IUI**							

LBR = 8.7%	Goverde et al ^13^	£10,400	£10,900	£11,600	£12,300	£13,000	£13,800
LBR varying by cycle order*	Local unit's experience and peer-reviewed literature ^6–10^	£7,700	£12,300	£16,800	£19,900	£24,300	£28,300
							

**U-IUI**							

LBR = 4.4%	LBR of U-IUI as half of that of S-IUI^11^	£0	£7,500	£8,700	£9,300	£9,800	£10,200
LBR = 2%	Local experience and expert opinion	¶	£91,700	£42,400	£35,700	£33,300	£32,100
LBR varying by cycle order±	LBR of U-IUI as half of that of S-IUI^11^	£41,600	£40,400	£47,600	£51,900	£54,900	£57,100
							

**C-IUI**							

LBR = 6.6%	Reasonable assumption, LBR of C-IUI between the values of LBR of U-IUI and S-IUI	£9,500	£10,500	£10,700	£10,800	£10,900	£10,900
LBR = 2.5%	Local experience and expert opinion	¶	£81,900	£53,900	£46,900	£43,700	£41,900

* LBR of S-IUI of 10%, 6%, 4%, 4%, 2%, 2% for cycles 1 to 6 respectively

± LBR of U-IUI of 5%, 3%, 2% 2%, 2%, 2% for cycles 1 to 6 respectively.

¶It was not possible to estimate a meaningful ICER because of very small denominator in case of one cycle of U-IUI (when LBR = 2%) and one cycle of C-IUI (when LBR = 2.5%) as compared to 1 full cycle of IVF.

ICER was estimated based on cost and effectiveness of "IUI+IVF" compared to full cycle of IVF.

Figures are rounded to the nearest hundred

**Table 5:** Sensitivity analysis estimates of cost difference, and the additional number of live birth-producing pregnancies by the estimated cost difference of providing "IUI+IVF" compared to primary offer of IVF, varied sequentially.

**Variable varied**	**Cost difference £ [("IUI+IVF") - IVF]**					
**S-IUI**	**1 cycle of IUI**	**2 cycles of IUI**	**3 cycles of IUI**	**4 cycles of IUI**	**5 cycles of IUI**	**6 cycles of IUI**

LBR = 8.7%	£43,100	£111,900	£183,400	£257,400	£333,800	£412,200
LBR varying by cycle order*	£39,400	£114,200	£199,500	£287,200	£379,900	£474,400
						

**U-IUI**						

LBR = 4.4%	£0	£30,700	£62,100	£94,100	£126,600	£159,700
LBR = 2%	£6,900	£45,300	£83,700	£122,300	£161,000	£199,900
LBR varying by cycle order±	£53,800	£141,100	£233,400	£326,800	£420,300	£514,000

**C-IUI**						

LBR = 6.6%	£23,900	£76,300	£128,900	£181,600	£234,200	£286,800
LBR = 2.5%	£35,800	£102,100	£168,400	£234,700	£301,000	£367,300
						

	**No. of live births expected from full cycles of IVF that could be purchased by the estimated cost difference**					

**S-IUI**						

LBR = 8.7%	3	9	15	20	27	33
LBR varying by cycle order*	3	9	16	23	30	38
						

**U-IUI**						

LBR = 4.4%	0	2	5	7	10	13
LBR = 2%	1	4	7	10	13	16
LBR varying by cycle order±	4	11	19	26	33	41
						

**C-IUI**						

LBR = 6.6%	2	6	10	14	19	23
LBR = 2.5%	3	8	13	19	24	29

* LBR of S-IUI of 10%, 6%, 4%, 4%, 2%, 2% for cycles 1 to 6 respectively

± LBR of U-IUI of 5%, 3%, 2% 2%, 2%, 2% for cycles 1 to 6 respectively.

Figures of cost differences are rounded to the nearest hundred.
