# Supplementary material for: Cost-effectiveness of primary offer of IVF vs. primary offer of IUI followed by IVF (for IUI failures) in couples with unexplained or mild male factor subfertility
Source: BMC Health Serv Res. 2006 Jun 23;6:80. doi: 10.1186/1472-6963-6-80 (PMC1543624; doi:10.1186/1472-6963-6-80)
Supplement: Additional File 1 — EVEREST Statement: Checklist for health economics paper. EVEREST checklist for health economics studies with respect to this study design, data collection, and analysis and interpretation of the results [file 1472-6963-6-80-S1.doc]

Additional file 1

#### EVEREST Statement: Checklist for health economics paper

|  | Study section | Additional remarks |
| --- | --- | --- |
| Study design |  |  |
| (1) The research question is stated | Background |  |
| (2) The economic importance of the research question is stated | Background |  |
| (3) The viewpoint(s) of the analysis are clearly stated and justified | Methods-Cost-effectiveness; Discussion |  |
| (4) The rationale for choosing the alternative programmes or interventions compared is stated | Background |  |
| (5) The alternatives being compared are clearly described | Background; Methods-Model construction |  |
| (6) The form of economic evaluation used is stated | Background; Methods-Cost-effectiveness |  |
| (7) The choice of form of economic evaluation is justified in relation to the questions addressed | Background; Discussion |  |
|  |  |  |
| Data collection |  |  |
| (8) The source(s) of effectiveness estimates used are stated | Methods-Assumptions used in the model; Table 1; Table 4 |  |
| (9) Details of the design and results of effectiveness study are given (if based on single study) | N/A | Data derived from HFEA database, peer reviewed literature, local unit experience, expert opinion |
| (10) Details of the method of synthesis or meta-analysis of estimates are given (if based on an overview of a number of effectiveness studies) | Methods-Assumptions used in the model | LBR for U-IUI and C-IUI were derived from meta-analyses comparing these LBR to that of S-IUI |
| (11) The primary outcome measure(s) for the economic evaluation are clearly stated | Methods-Model construction |  |
| (12) Methods to value health states and other benefits are stated | N/A |  |
| (13) Details of the subjects from whom valuations were obtained are given | N/A |  |
| (14) Productivity changes (if included) are reported separately | N/A |  |
| (15) The relevance of productivity changes to the study question is discussed | N/A |  |
| (16) Quantities of resources are reported separately from their unit costs | Methods-Cost calculations; Table 1 | Nominal cost of IVF and IUI are derived from the economic model used by NICE |
| (17) Methods for the estimation of quantities and unit costs are described | Methods-Cost calculations; Table 1 | Costs were not synthesised from individual units. Nominal cost of IVF and IUI were derived from the economic model used by NICE |
| (18) Currency and price data are recorded | Methods-Cost calculations; Tables 1- 5 |  |
| (19) Details of currency of price adjustments for inflation or currency conversion are given | NA | As the study is looking for relative cost, then inflation would be comparable between the different treatments |
| (20) Details of any model used are given | Methods-Model construction |  |
| (21) The choice of model used and the key parameters on which it is based are justified | Methods-Model construction |  |
|  |  |  |
| Analysis and interpretation of results |  |  |
| (22) Time horizon of costs and benefits is stated | Methods-Model construction; Discussion | Based on current cost estimates |
| (23) The discount rate(s) is stated | N/A |  |
| (24) The choice of rate(s) is justified | N/A |  |
| (25) An explanation is given if costs or benefits are not discounted | N/A |  |
| (26) Details of statistical tests and confidence intervals are given for stochastic data | N/A |  |
| (27) The approach to sensitivity analysis is given | Methods-Sensitivity analysis |  |
| (28) The choice of variables for sensitivity analysis is justified | Methods-Sensitivity analysis; Table 4 |  |
| (29) The ranges over which the variables are varied are stated | Table 4 |  |
| (30) Relevant alternatives are compared | Methods-Model construction |  |
| (31) Incremental analysis is reported | Methods-Cost-effectiveness; Table 2; Table 4 |  |
| (32) Major outcomes are presented in a disaggregated as well as aggregated form | Table 2; Table 3 |  |
| (33) The answer to the study question is given | Discussion; Conclusion |  |
| (34) Conclusions follow from the data reported | Conclusion |  |
| (35) Conclusions are accompanied by the appropriate caveats | Discussion; Conclusion |  |
