# Supplementary material for: Cost-effectiveness of primary offer of IVF vs. primary offer of IUI followed by IVF (for IUI failures) in couples with unexplained or mild male factor subfertility
Source: BMC Health Serv Res. 2006 Jun 23;6:80. doi: 10.1186/1472-6963-6-80 (PMC1543624; doi:10.1186/1472-6963-6-80)
Supplement: Additional File 2 — Local data on outcome of 676 cycles of S-IUI in 334 couples with unexplained infertility women aged <40 years. Results of local data on S-IUI uptake and outcome, live birth-producing pregnancy, by treatment cycle order. [file 1472-6963-6-80-S2.doc]

Additional File 2

Local data on outcome of 676 cycles of S-IUI in 334 couples with unexplained infertility women aged <40 years [4].

| Treatment Cycle | Number not pregnant at start of cycle | Live birth during cycle | Lost to follow- up at end of interval | Proportion with live birth in interval | Cumulative proportion live birth |
| --- | --- | --- | --- | --- | --- |
| 1 | 334 | 34 | 83 | 0.10 | 0.10 |
| 2 | 217 | 7 | 123 | 0.03 | 0.13 |
| 3 | 97 | 5 | 57 | 0.05 | 0.18 |
| 4 | 25 | 1 | 21 | 0.04 | 0.21 |
| 5 | 3 | 0 |  | 0.00 | 0.21 |
